# Gonorrhoeal Rheumatism

**Published:** 1891-01

**Authors:** 


### GONORRHŒAL RHEUMATISM.

Prof. Peter, in his clinical lectures at the Necker Hospital, has been showing a number of these cases, in which he was able to demonstrate his theory of the causes of this disease, and prove that his treatment was efficacious.

The fact that such cases are seen without any gonorrhœa does away with the idea that the disease is properly named gonorrhœal rheumatism, and it often originates in any inflammatory discharge connected with the urethra. Dr. Peter believes that this trouble is due entirely to reflex disturbances. The urethral imflammation affects certain centers in the spinal cord and brain, and the altered conditions of these give rise to the changes in the articulation; and it is very reasonable to suppose that this is the correct theory in regard to this complaint. The male urethra, with all its complex and sensitive actions, its miction, its function of erection, and ejaculation, show that it is an organ that is highly sensitive, and one that would induce reflex action more than any other in the human body. The very fact that women enjoy a comparative immunity from gonorrhœal rheumatism proves this theory, because the urethra of woman is used for miction only, and has none of the compound functions that the male one has, and is therefore less liable to reflex action. Be this theory correct or not, we wish simply in this short abstract of Professor Peter's lecture to call attention to his successful treatment used for such cases. The usual drug administration being most unsatisfactory, alkalies, salines, iodide of potassium, quinine, iron, etc., give little or no results, and the cases usually finish up with a permanent form of anchylosis, no matter what is done. Dr. Peter, however, has been able to show that three things will avert this ending, and these are revulsion, massage and immobility. The attention of the careful practitioner must only be directed to the local manifestations, and the patient must be kept in bed while the affected joint is protected by an apparatus in such a way that it shall be immobile, and then active revulsion must be applied at first with thermo cautery points up to *five and six hundred on each joint;* after this massage until the patient can walk. We have seen this active treatment bring around several of Dr. Peter's patients with the ability to walk, and rescue them from anchylosis. The blistering is very painful, but no anodynes are needed, as they only retard a cure. Dr. Peter says that poultices, opiated or not, and ointments containing drugs, only condemn the patient to certain anchylosis. The administration of internal remedies is but little better, unless there is a syphilitic taint, when mercurials and iodide of potassium may assist a cure; but revulsion of an energetic type will be the only sure prevention of ancholysis, assisted by massage, to be continued for a long time. Sodium salicylate is useless.—*Times and Register.*

